# Interferon-lambda1 induces peripheral blood mononuclear cell-derived chemokines secretion in patients with systemic lupus erythematosus: its correlation with disease activity

**DOI:** 10.1186/ar3363

**Published:** 2011-06-16

**Authors:** Qian Wu, Qingrui Yang, Elaine Lourenco, Hongsheng Sun, Yuanchao Zhang

**Affiliations:** 1 Department of Rheumatology, Provincial Hospital Affiliated to Shandong University, 324 Jing Wu Road, Jinan, 250021, People's Republic of China; 2 Division of Rheumatology, David Geffen School of Medicine, University of California Los Angeles, 1000 Veteran Avenue, Los Angeles, CA, 90095, USA

## Abstract

**Introduction:**

Systemic lupus erythematosus (SLE) is an autoimmune disease involving multiple organ systems. Previous studies have suggested that interferon-lambda 1 (IFN-λ1), a type III interferon, plays an immunomodulatory role. In this study we investigated its role in SLE, including its correlation with disease activity, organ disorder and production of chemokines.

**Methods:**

We determined levels of IFN-λ1 mRNA in peripheral blood mononuclear cells (PBMC) and serum protein levels in patients with SLE using real-time polymerase chain reaction (real-time PCR) and enzyme-linked immunoassay (ELISA). Further, we detected the concentration of IFN-inducible protein-10 (IP-10), monokine induced by IFN-γ (MIG) and interleukin-8 (IL-8) secreted by PBMC under the stimulation of IFN-λ1 using ELISA.

**Results:**

IFN-λ1 mRNA and serum protein levels were higher in patients with SLE compared with healthy controls. Patients with active disease showed higher IFN-λ1 mRNA and serum protein levels compared with those with inactive disease as well. Serum IFN-λ1 levels were positively correlated with Systemic Lupus Erythematosus Disease Activity Index (SLEDAI), anti-dsDNA antibody, C-reactive protein (CRP) and negatively correlated with complement 3. Serum IFN-λ1 levels were higher in SLE patients with renal involvement and arthritis compared with patients without the above-mentioned manifestations. IFN-λ1 with different concentrations displayed different effects on the secretion of the chemokines IP-10, MIG and IL-8.

**Conclusions:**

These findings indicate that IFN-λ1 is probably involved in the renal disorder and arthritis progression of SLE and associated with disease activity. Moreover, it probably plays an important role in the pathogenesis of SLE by stimulating secretion of the chemokines IP-10, MIG and IL-8. Thus, IFN-λ1 may provide a novel research target for the pathogenesis and therapy of SLE.

## Introduction

Systemic lupus erythematosus (SLE) is an autoimmune and inflammatory disease characterized by the activation of T and polyclonal B lymphocytes, production of numerous autoantibodies, and formation of immune complexes that result in tissue and organ damage [1].

The interferon (IFN) family plays an important role in innate as well as adaptive immune responses against viral infections [2]. Classic IFNs include the type I subgroup composed of IFN-α, IFN-β, IFN-ω, IFN-κ, IFN-τ, and the type II subgroup represented by IFN-γ [3]. Previous studies have suggested that both subgroups play an important role in the pathogenesis of SLE [3-6].

Type III IFN, IFN-λ1, IFN-λ2, IFN-λ3, also referred to as interleukin (IL)-29, IL-28A and IL-28B, respectively, are novel members of the IFN super-family [7,8]. They are secreted by human peripheral blood mononuclear cells (PBMC) as well as dendritic cells (DC) upon infection with viruses or stimulation with poly (I:C) or lipopolysaccharide (LPS) [2,8], and express in a broad spectrum of tissues [7]. Gene expressions are regulated by virus-activated interferon regulatory factor (IRF) 3 and IRF 7 [9]. These proteins induce activation of JAK/STAT signaling pathways through a cell-surface receptor consisting of two chains, IFN-λR1, which is IFN-λ- specific, and IL-10R2, which is shared among IL-10, IL-22 and IL-26 [10,11].

IFN-λ share several common features with type I IFN, such as antiviral, anti-proliferative as well as antitumor activities [2,10,12-15], meantime, their immune-regulatory function has gradually been elucidated as well. Recent studies have reported that IFN-λ-treated DC specifically induced proliferation of a CD4+CD25+Foxp3+T cell subset [16]. IFN-λ1 was able to inhibit human type 2 helper T (Th2) cell responses by diminishing secretion of IL-13, and also specifically upregulated cytokines IL-6, IL-8 and IL-10 levels secreted by monocytes in a dose-dependent manner [17-19].

Chemokines are a group of small molecules with the ability to direct cell movements necessary for the initiation of T cell immune response, recruit specific leucocytes to inflammatory sites, regulate polarization of Th1 and Th2 lymphocytes, and influence maturation of DC, T cell and bone marrow progenitor [20-23]. Moreover, they can stimulate monocytes, natural killer (NK) and T cell migration, and modulate adhesion molecule expansion [24]. Therefore, they are related to tissue inflammation and organ damage in SLE.

IFN-inducible protein-10 (IP-10) is a CXC chemokine secreted by PBMC, fibroblasts and endothelial cells [25], and plays an important role in the perpetuation of chronic inflammatory responses by promoting the recruitment of monocytes, T and NK cells into target tissue and organ [26]. IL-8, another CXC chemokine, is predominantly chemotactic for neutrophils, and also has the capability of recruiting leukocytes to the glomerulus during immune renal damage [27].

Many studies have discovered that plasma chemokine concentrations including IL-8, IP-10 and monokine induced by IFN-γ (MIG) are elevated in patients with active SLE [28-31]. Some studies have reported that urinary IL-8 levels are increased in SLE patients with active renal disease as well [27,32].

With this background, we compared expression of IFN-λ1 mRNA in PBMC and serum protein levels in SLE patients with healthy controls. In addition, we determined the correlation of serum IFN-λ1 levels with disease activity and clinical manifestations in SLE, and investigated the effect of IFN-λ1 on the secretion of the chemokines IP-10, MIG and IL-8.

## Materials and methods

### Patients and controls individuals

This study was approved by the Review Board for Shandong Provincial Hospital in Jinan, People's Republic of China. Informed consent was obtained from all study participants. A total of 42 patients meeting the revised American College of Rheumatology criteria for SLE and 25 age-matched and sex-matched healthy controls were enrolled in the present study. All SLE patients were recruited from the Rheumatology Department, Provincial Hospital Affiliated to Shandong University, and individuals with other rheumatic diseases, infections or malignant tumors were excluded from the study. Healthy controls were selected from a great many healthy volunteers at the Provincial Hospital Affiliated to Shandong University in order to match them to the SLE patients in terms of age and sex.

SLE patients' laboratory tests containing anti-double stranded (ds) DNA antibody, anti-nucleosome antibody (AnuA), anti-smith-antibody, anti- ribosome ribonucleoprotein antibody (rRNP), anti-histone antibody (AHA), erythrocyte sedimentation rate (ESR), C-reactive protein (CRP), complement 3 (C3) and C4 as well as 24-hour urine protein were performed. Clinical data from each patient were recorded. These were new patients who were diagnosed with SLE for the first time and needed to receive steroid therapy with an average prednisone (or equivalent) dosage of 10 mg/day (median 10 mg, range 5 to 15 mg) according to their disease condition at that time. Before their blood samples were prepared, 26 patients had not taken prednisone, and 16 patients had taken prednisone once. Lupus disease activities were assessed using the Systemic Lupus Erythematosus Disease Activity Index (SLEDAI) score [33]. Active lupus disease was defined as a SLEDAI score ≥6 [33]. Characteristics of the SLE patients and healthy controls are listed in Table 1.

**Table 1:** Demographics of SLE and healthy controls

	SLE patients (*n *= 42)	Healthy donors (*n *= 25)
Age (years)	27.4 ± 10.12 (13 to 45)	25.2 ± 9.58 (20 to 42)
Sex(female/male)	39/3	23/2
Disease duration(years)	2.45 ± 2.67	-
Alopecia n (%)	16 (38.1)	-
Mucosal ulcer n (%)	10 (23.8)	-
Malar rash n (%)	26 (61.9)	-
Arthritis n (%)	29 (69.1)	-
Current renal disease n (%)	25 (59.5)	-
Pleuritis n (%)	2 (4.8)	-
Fever n (%)	10 (23.8)	-
Neurological disorder n (%)	2 (4.8)	-
Anemia n (%)	12 (28.6)	-
Thrombocytopenia n (%)	8 (19)	-
Leukopenia n (%)	22 (52.4)	-
ds-DNA n (%)	26 (61.9)	-
AnuA n (%)	20 (47.6)	-
Smith n (%)	10 (23.8)	-
AHA n (%)	20 (47.6)	-
rRNP n (%)	8 (19)	
ESR	34.05 ± 26.79	-
CRP	5.31 ± 6.44	-
Low C3 n (%)	26 (61.9)	-
Low C4 n (%)	22 (52.4)	-
24-hour urine protein n (%)	22 (52.4) (> 0.5 g/24 h)	-
SLEDAI	4 to 30 (13.9 ± 7.12)	-

Except where otherwise indicated, values are expressed as mean ± standard deviation. There were no significant differences between patients with SLE and healthy donors in terms of age and sex. AHA, anti-histone antibody; AnuA, anti-nucleosome antibody; C3, complement 3; C4, complement 4; CRP, C-reactive protein; ds-DNA, anti-double stranded DNA antibody; ESR, erythrocyte sedimentation rate; rRNP, anti-ribosome ribonucleoprotein antibody; SLE, systemic lupus erythematosus; SLEDAI, SLE disease activity index; Smith, anti-smith-antibody.

### Blood samples

Fasting venous blood (4 ml) was collected and processed within two hours. PBMC were isolated from patients and healthy controls by density-gradient centrifugation over 'Histopaque-1077' (Sigma, St Louis, MO, USA) for cell culture or stored at -80°C until RNA extraction. Serum samples were stored at -80°C until cytokine were determined.

### RNA extraction

Total RNA was extracted from PBMC with Trizol (Invitrogen, Carlsbad, CA, USA) according to the manufacturer's instructions. Then the quantity and purity of RNA was determined by absorbance on a spectrophotometer (Beckman Instruments, Fullerton, CA, USA) at 260 nm and 280 nm. Samples with ratios from 1.8 to 2.0 were accepted for next reverse transcription reaction.

### Reverse transcription reaction

The 20-μl cDNA synthesis reaction was performed with 0.3 μg RNA containing 1 μl of random hexamer primers, 4 μl of 5 × reaction buffer, 2 μl of 10 mM dNTP mix, and 1 μl of RiboLock ribonuclease inhibitor (Fermentas, Burlington, Ontario, Canada. Reverse transcription was carried out at 25°C for 10 minutes, 42°C for 60 minutes, and 70°C for 10 minutes using Gene Amp PCR system 9700 (Applied Biosystems, Foster City, CA, USA).

### Real-time polymerase chain reaction

The primers were designed by BIOSUNE (Shanghai, China): IFN-λ1 forward primer 5'-TAT CCA GCC TCA GCC CAC AG-3', reverse primer 5'-CTC AGA CAC AGG TTC CCA TCG-3'; β-actin forward primer 5'-CAC TCT TCC AGC CTT CCT TCC-3', reverse primer 5'-AGG TCT TTG CGG ATG TCC AC-3'. Real-time polymerase chain reaction (PCR) amplification reactions were prepared with the SYBR Green PCR Master Mix (Applied Biosystems, USA) and performed using the 7500 Real-Time PCR system (Applied Biosystems, USA). Each 20 μl real-time PCR included 10 μl of SYBR Green PCR Master Mix, 5 μl of primers (concentrations were 0.2 μmol) and 5 μl of cDNA (after reverse transcription diluted 1:5 with Diethyl Pyrocarbonate water). PCR conditions consisted of initial denaturation at 95°C for 10 minutes, followed by 40 cycles of denaturation at 95°C for 15 s, and annealing extension at 60°C for 1 minute. PCR products were verified by melting curve analysis. Relative mRNA levels were determined by the 2^**-ΔΔct **^method.

### Cell culture condition

Culture medium, consisted of RPMI 1640 medium (Hycolne, Logan, UT, USA) supplemented with 10% Fetal Calf Serum (Gibco-Invitrogen, Mulgrave, Victoria, Australia), 2 mmol/L L-glutamine, 100 IU/mL penicillin and 100 μg/mL streptomycin (Sigma, Ronkonkoma, NY, USA), respectively. Whole PBMC were cultured in 24-well, flat-bottomed plates (5 × 10^5 ^in 1 ml) for 72 h. In the PBMC culture system there were different culture groups: PBMC alone, PBMC were stimulated with LPS at 100 ng/ml (Sigma, USA), PBMC were stimulated with human recombinant IFN-λ1 at 10 ng/ml, 50 ng/ml, 100 ng/ml (Peprotech, Rocky Hill, NJ, USA), respectively, PBMC were stimulated with LPS at 100 ng/ml in the presence of human recombinant IFN-λ1 at 10 ng/ml, 50 ng/ml and 100 ng/ml, respectively. In the experiment of observing the synergistic effect of IFN-λ1 and LPS, whole PBMC were incubated in the presence of different concentration of IFN-λ1 for 30 minutes before the addition of LPS.

Supernatants were harvested and froze at -80°C for later cytokine analysis by ELISA.

### Enzyme-linked immunosorbent assay

Serum IFN-λ1 levels and cell culture supernatant MIG, IP-10 and IL-8 levels were determined by enzyme-linked immunosorbent assay (ELISA) following the manufacturer's instructions. IFN-λ1 was quantified using ELISA reagent kits purchased from Adlitteram Diagnostic Laboratories (San Diego, CA, USA). Detection of the chemokines MIG, IP-10 and IL-8 was accomplished using the Bender MedSystem (Vienna, Austria)

### Statistical analysis

Differences in IFN-λ1 mRNA expression and serum protein levels as well as differences of chemokines MIG, IP-10 and IL-8 levels among the different populations were determined by Mann-Whitney U-test, one-way ANOVA with Bonferroni analysis. Spearman correlation test was used to assess the association between serum IFN-λ1 levels and different variables. Analysis was performed with Statistical Package for the Social Science (SPSS) version 16.0. (SPSS Inc., Chicago, IL, USA). *P *< 0.05 was considered statistically significant.

## Results

### IFN-λ1 mRNA and serum protein levels were higher in patients with SLE compared with healthy controls

**Figure 1:**
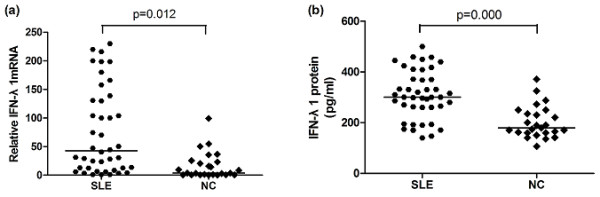
**Comparison of IFNλ1 mRNA and protein levels between SLE and NC**. The methods employed to detect expression levels of IFNλ1 mRNA and protein levels are described in Materials and methods. (**a**) IFNλ1 mRNA levels were significantly elevated in SLE patients versus NC. (**b**) Serum IFNλ1 protein levels were significantly elevated in SLE patients versus NC. Each symbol represents an individual patient and healthy donor. Horizontal lines indicate median values. IFNλ1, interferon λ1; NC, normal control; SLE, systemic lupus erythematosus.

Initially, the expression of IFN-λ1 mRNA in PBMC and serum IFN-λ1 protein levels from 42 SLE patients and 25 normal controls (NC) were measured using real-time reverse transcription PCR and ELISA, respectively. SLE patients and normal controls did not reveal significant differences in terms of mean age or sex distribution (Table 1). As shown in Figure 1a, SLE patients had significantly higher IFN-λ1 mRNA level than did normal controls (*P *= 0.012). Figure 1b also displayed significant elevation of serum IFN-λ1 protein levels in patients with SLE compared with normal controls (*P *= 0.000), indicating that IFN-λ1 probably participated in the pathogenesis of SLE.

### IFN-λ1 mRNA and serum protein levels were higher in SLE patients with active disease compared with those with inactive disease

**Figure 2:**
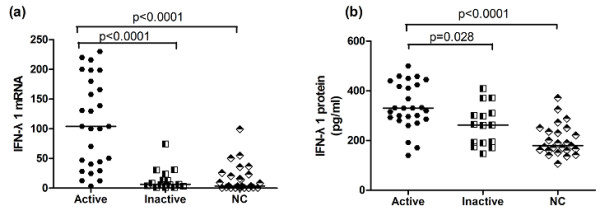
**Comparison of IFNλ1 mRNA and protein levels among SLE patients with active disease and inactive disease as well as NC**. (**a**) IFNλ1 mRNA levels were significantly elevated in SLE patients with active disease (*n *= 27) compared with those with inactive disease (*n *= 15) as well as NC. (**b**) Serum IFNλ1 protein levels were significantly elevated in SLE patients with active disease compared with those with inactive disease together with NC. Each symbol represents an individual patient. horizontal lines indicate median values. IFNλ1, interferon λ1; NC, normal control; SLE, systemic lupus erythematosus.

We next investigated whether IFN-λ1 was related to disease activity in SLE patients. We divided SLE patients into active groups (SLEDAI score ≥6) and inactive groups (SLEDAI score < 6) according to SLEDAI. As seen in Figure 2a, b, significant differences were viewed in IFN-λ1 mRNA and protein levels between patients with active and those with inactive disease (*P *< 0.0001, *P *= 0.028). In the meantime, patients with active disease displayed higher IFN-λ1 mRNA and serum protein levels compared with normal controls (*P *< 0.0001, *P *< 0.0001); however, we did not observe the differences of IFN-λ1 mRNA and protein levels between patients with inactive disease and normal controls (data not shown). Thus, we speculated that IFN-λ1 probably was associated with disease activity in SLE.

### Correlation between IFN-λ1 levels and SLEDAI as well as laboratory values

**Figure 3:**
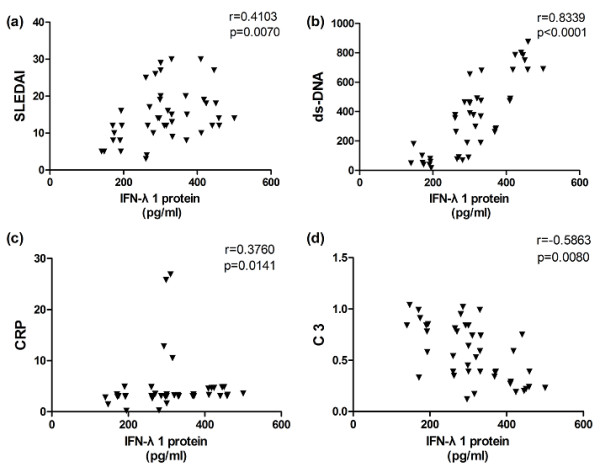
**Association of serum IFN-λ1 levels with SLEDAI as well as laboratory values**. Each symbol represents an individual patient. (**a**) Serum IFN-λ1 levels were positively correlated with SLEDAI. (**b**) A significantly positive correlation was observed between serum IFN-λ1 levels and anti-dsDNA antibody. (**c**) Positive correlation was also seen between serum IFN-λ1 levels and CRP. (**d**) Negative relationship was observed between serum IFN-λ1 levels and C3. anti-dsDNA antibody, anti-double stranded DNA antibody; C3, complement 3; CRP, C-reactive protein; IFNλ1, interferon λ1; SLEDAI, systemic lupus erythematosus disease activity index.

To further survey the relationship between serum IFN-λ1 protein levels and disease activity, we next determined correlations between IFN-λ1 and SLEDAI as well as laboratory values containing anti-dsDNA, AnuA, smith, rRNP, AHA antibody, ESR, CRP, C3, C4 and 24-hour urine protein. We surveyed that serum IFN-λ1 protein levels were positively correlated with SLEDAI, anti-dsDNA antibody and CRP (r = 0.4103, *P *= 0.007, Figure 3a; r = 0.8339, *P *< 0.0001, Figure 3b; r = 0.3760, *P *= 0.0141, Figure 3c). There was a negative correlation between serum IFN-λ1 levels and complement C3 (r = -0.5863, *P *= 0.008, Figure 3d). No significant correlations were found between serum IFN-λ1 levels and anti -AnuA, smith, rRNP, AHA antibody, ESR, C4 and 24-hour urine protein (Table 2).

**Table 2:** Correlation between IFN-λ1 levels and SLEDAI as well as laboratory values

Parameter	Correlation coefficient (*r*)	*P*-value
SLEDAI	0.4103	0.007
dsDNA	0.8339	< 0.0001
AnuA	0.598	0.133
Smith	0.798	0.063
rRNP	0.091	0.399
AHA	0.414	0.219
ESR	0.536	0.475
CRP	0.376	0.0141
C3	-0.5863	0.008
C4	0.049	0.093
24-hour urine protein	0.759	0.436

AHA, anti-histone antibody; AnuA, anti-nucleosome antibody; C3, complement 3; C4, complement 4; CRP, C-reactive protein; ds-DNA, anti-double stranded DNA antibody; ESR, erythrocyte sedimentation rate; rRNP, anti-ribosome ribonucleoprotein antibody; SLEDAI, SLE disease activity index; Smith, anti-smith-antibody.

### Association of serum IFN-λ1 protein levels with clinical features in SLE

**Figure 4:**
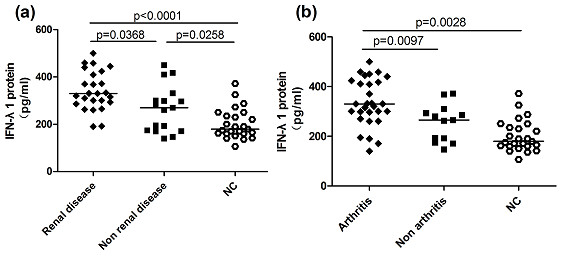
**Elevated serum IFN-λ1 levels in SLE patients with organ damage**. (**a**) Serum IFN-λ1 levels exhibited a significant elevation in patients with renal involvement (*n *= 25) relative to patients without renal involvement (*n *= 17) as well as NC; patients in the absence of renal disorder also displayed higher serum IFN-λ1 levels compared with NC. (**b**) Serum IFN-λ1 levels were significantly higher in patients in the presence of arthritis (*n *= 29) than patients in the absence of arthritis (*n *= 13) as well as NC. Each symbol represents an individual patient; horizontal lines indicate median values. IFNλ1, interferon λ1; NC, normal control; SLE, systemic lupus erythematosus.

To assess associations between serum IFN-λ1 protein levels and clinical manifestations, serum IFN-λ1 protein levels were compared among patients with and those without certain clinical features as well as normal controls. We identified that no significant differences in serum IFN-λ1 protein levels between patients in the presence of alopecia, mucosal ulcer, malar rash, chest affection, fever, neurological disorder, anemia, thrombocytopenia, leucopenia and patients in the absence of the above-mentioned clinical manifestations (Table 3). Nevertheless, we discerned that serum IFN-λ1 levels were significantly higher in patients with renal disease and arthritis compared with patients without these manifestations together with normal controls. (*P *= 0.0368, *P *< 0.0001, Figure 4a; *P *= 0.0097, *P *= 0.0028, Figure 4b), meantime, patients in the absence of renal disorder had higher serum IFN-λ1 level compared with normal controls as well (*P *= 0.0258, Figure 4a), but patients without arthritis did not show significant higher serum IFN-λ1 level than normal controls (data not shown), illustrating that IFN-λ1 probably acted in the development of renal disorder and arthritis in SLE.

**Table 3:** Serum IFN-λ1 protein by presence or absence of SLE clinical characteristics

Clinical characteristics	Present n Median (interquartile range)	Absent n Median (interquartile range)	*P*- value
**Alopecia**	16 297.839 (415.787 to 193.064)	26 305.489 (368.886 to 267.957)	NS
**Mucosal ulcer**	10 307.276 (432.756 to 194.393)	32 300.582 (369.881 to 261.142)	NS
**Malar rash**	26 299.715 (381.168 to 245.771)	16 310.309 (400.762 to 260.412)	NS
**Fever**	10 300.520 (340.477 to 237.890)	32 305.487 (410.534 to 263.343)	NS
**Chest affection**	2 385.455 (440.400 to 330.510)	40 300.522 (371.396 to 260.412)	NS
**Renal disease**	25 330.060 (416.827 to 289.660)	17 270.400 (315.764 to 172.973)	0.0368
**Neurological disorder**	2 246.952 (298.846 to 195.058)	40 305.488 (400.033 to 261.142)	NS
**Arthritis**	29 330.060 (421.041 to 283.616)	13 265.347 (301.716 to 183.47)	0.0097
**Anemia**	12 307.276 (422.614 to 211.962)	30 300.582 (368.886 to 260.557)	NS
**Thrombocytopenia**	8 300.520 (330.398 to 192.99)	34 305.488 (409.822 to 262.164)	NS
**Leucopenia**	22 308.144 (411.573 to 245.771)	20 300.520 (360.412 to 260.412)	NS

NS, not significant.

### IFN-λ1 induced IP-10, MIG and IL-8 production in PBMC of SLE patients

**Figure 5:**
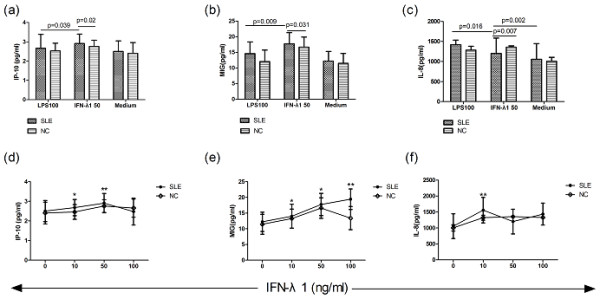
**IFN-λ1-induced chemokines production by human PBMC and dose-relationship of IFN-λ1- induced chemokines by PBMC**. Human PBMC were cultured for 72 h in the presence of recombinant IFN-λ1 and supernatants were examined for levels of IP-10, MIG and IL-8 using ELISA. The chemokines' response to LPS was also examined as a positive control. In SLE patients, IFN-λ1-stimulated PBMC displayed higher levels of chemokines IP-10 (**a**) and MIG (**b**) in comparison with positive control LPS. IFN-λ1 could induce more generation levels of chemokines IP-10 (a) and MIG (b) in SLE patients than normal controls, but the secretion levels of IL-8 were lower in SLE patients than normal controls (**c**). Meanwhile, in patients with SLE, IFN-λ1 induced less secretion of chemokine IL-8 than LPS, but it had the ability to stimulate more IL-8 production than medium (c). Then human PBMC were cultured over different IFN-λ1 concentrations for 72 h and supernatants were examined for IP-10 (**d**), MIG (**e**) and IL-8 (**f**) levels using ELISA. Both 10 ng/ml and 50 ng/ml IFN-λ1 had effects on the secretion of IP-10 (*P *= 0.033, 0.005) (d). IFN-λ1 displayed its effects at the three different concentration on MIG secretion with dose-dependent relation (*P *= 0.043, 0.016, 0.001) (e). Compared with other two concentration, 10 ng/ml IFN-λ1 had the most obvious effect in the secretion of IL-8 (*P *= 0.008) (f). *, *P *< 0.05, **, *P *< 0.01. means ± s.d. are shown. ELISA, enzyme-linked immunosorbent assay; LPS, lipopolysaccharide; IFNλ1, interferon λ1; IL-8, interleukin-8; IP-10, IFN-inducible protein-10; MIG, monokine induced by interferon-γ; NC, normal control; PBMC, peripheral blood mononuclear cells; SLE, systemic lupus erythematosus.

Next, we inquired into whether IFN-λ1 played a role in the secretion of several chemokines involved in the pathogenesis of SLE. We approached the chemokines produced by SLE patients PBMC in response to IFN-λ1 and contrasted it to that obtained following LPS stimulation. As manifested in Figure 5, in both SLE patients and normal controls, IFN-λ1-stimulated PBMC emerged higher levels of chemokines IP-10 (*P *= 0.039, 0.028, Figure 5a) and MIG (*P *= 0.009, 0.038, Figure 5b) in comparison with positive control LPS, and about the secretion of IL-8, the same result was observed in normal controls (*P *= 0.049, Figure 5c). Moreover, at 50 ng/ml IFN-λ1, the generation levels of chemokines IP-10 and MIG in SLE patients were more than normal controls (*P *= 0.02, Figure 5a; *P *= 0.031, Figure 5b), and the secretion levels of IL-8 were lower in SLE patients than normal controls (*P *= 0.007, Figure 5c). Meanwhile, in patients with SLE, IFN-λ1 induced less secretion of chemokine IL-8 than LPS (*P *= 0.016, Figure 5c), but it had the ability to stimulate more IL-8 production than medium (*P *= 0.002, Figure 5c). Thus, through the above observation, we can acquire the conclusion that in patients with SLE, IFN-λ1 was capable of inducing the production of chemokines IP-10, MIG and IL-8 which participate in the pathogenesis of SLE by their special mechanism.

We next chose to study the dose-response curves for IFN-λ1 inducing IP-10, MIG and IL-8, demonstrating that in SLE patients, both 10 ng/ml and 50 ng/ml IFN-λ1 had effects on the secretion of IP-10 (*P *= 0.033, 0.005, Figure 5d) and stimulated more IP-10 than normal controls (*P *= 0.024, 0.047, Figure 5d). IFN-λ1 displayed its effects at the three different concentration on MIG secretion with dose-dependent relation (*P *= 0.043, 0.016, 0.001, Figure 5e), and the secretion levels of MIG were higher compared with normal controls (*P *= 0.033, 0.029, 0.031, Figure 5e). Compared with other two concentration, 10 ng/ml IFN-λ1 had the most obvious effect in the secretion of IL-8, and stimulated more IL-8 secretion than normal controls (*P *= 0.008, 0.018, Figure 5f).

### IFN-λ1 enhanced the chemokines response to LPS in PBMC

**Figure 6:**
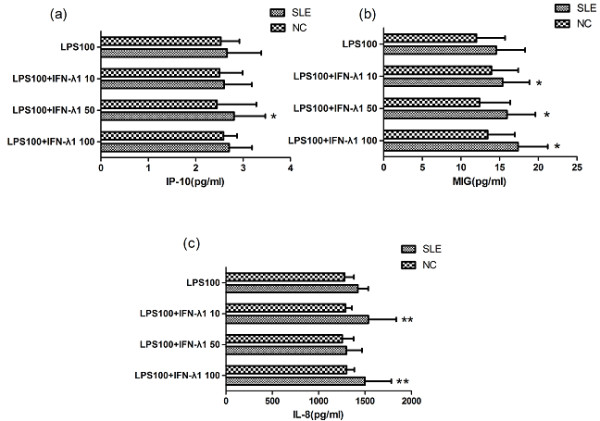
**IFN-λ1 increased the chemokines response to LPS**. Whole PBMC were incubated in the presence of IFN-λ1 for 30 minutes before the addition of LPS at the doses indicated. Cells were then incubated for 72 h before being assessed for levels of IP-10 (**a**), MIG (**b**) and IL-8 (**c**) in culture supernatants using ELISA. only 50 ng/ml IFN-λ1 showed an assistant effect on the chemokine IP-10 secretion (*P *= 0.016) (a). Different concentrations of IFN-λ1 all played a synergistic role in the effect of LPS (*P *= 0.047, 0.013, 0.038) (b). Both 10 ng/ml and 100 ng/ml IFN-λ1 displayed this synergistic effect on the secretion of chemokine IL-8 (*P *< 0.001, *P *= 0.002) (c). *, *P *< 0.05, **, *P *< 0.01. means ± s.d. are shown. ELISA, enzyme-linked immunosorbent assay; IFNλ1, interferon λ1; IL-8, interleukin-8; IP-10, IFN-inducible protein-10; LPS, lipopolysaccharide; MIG, monokine induced by interferon-γ; NC, normal control; PBMC, peripheral blood mononuclear cells; SLE, systemic lupus erythematosus.

We chose to examine IFN-λ1's ability to regulate chemokine response to LPS. In SLE patients, at 100 ng/ml LPS, only 50 ng/ml IFN-λ1 showed a synergistic effect on the chemokine IP-10 response (*P *= 0.016, Figure 6a). With regard to chemokine MIG, different concentration of IFN-λ1 all played an assistant role in the effect of LPS (*P *= 0.047, 0.013, 0.038, Figure 6b). Both 10 ng/ml and 100 ng/ml IFN-λ1 manifested this synergistic effect on the secretion of chemokine IL-8 when 100 ng/ml LPS were used (*P *< 0.001, *P *= 0.002, Figure 6c). In different culture condition, the generation levels of each chemokine were higher in SLE patients compared with normal controls (data not shown).

## Discussion

Systemic lupus erythematosus (SLE) is a chronic autoimmune disease affecting multiple organ systems, involving the skin, joints, heart, lungs, kidneys and central nervous system (CNS) [34]. Skin involvement, arthritis and renal disorder are very common manifestations in patients with SLE. Among them, skin involvement generally manifests as alopecia, mucosal ulcers or malar rash. Renal disorders range from asymptomatic hematuria or proteinuria to overt nephritic and nephrotic syndromes, rapidly progressive glomerulonephritis, and chronic renal failure [35]. So far, the pathogenesis of SLE has not been illuminated clearly.

Functions of IFN-λ1, a type III IFN, include inhibition of viral infection and proliferation of tumor cells as well as regulation of immune system [10,12-18,36]. To the best of our knowledge, the role of IFN-λ1 in the progression of SLE remains unknown. Therefore, we hypothesized that IFN-λ1 played a role in the pathogenesis of autoimmune diseases such as SLE. Real-time PCR and ELISA assays were used to detect IFN-λ1 mRNA expression in PBMC and serum IFN-λ1 protein levels, respectively. Our initial data showed that IFN-λ1 mRNA expression and serum protein levels in patients with SLE were higher compared with normal controls, suggesting a role for IFN-λ1 in the pathogenesis of SLE.

We were also interested as to whether IFN-λ1 was associated with disease activity. Under these circumstances, SLE patients were divided into active and inactive groups according to SLEDAI scores, and active groups were defined as a SLEDAI score ≥6 [33]. By analyzing the data of different groups, finding that IFN-λ1 mRNA and protein levels in patients with active disease were significantly higher compared with patients with inactive disease together with normal controls. Because we have known that IFN-λ1 can be secreted by PBMC as well as DC [2,8], meantime, SLE is a kind of autoimmune disease characterized by massive abnormal immune cells response which leads to autoimmune disorders and dysregulation. Therefore, we speculated that elevated IFN-λ1 mRNA and protein levels in patients with SLE probably were related to abundant and inordinate immune cell response. Thus, our findings implied that IFN-λ1 probably involved in the disease activity of SLE.

Moreover, we made further efforts to analyze the correlation between serum IFN-λ1 protein levels and SLEDAI together with several laboratory values, such as anti-dsDNA, AnuA, Smith, rRNP, AHA antibody, ESR, CRP, C3, C4 and 24-hour urine protein. Our analysis results disclosed that significant positive correlations were found between serum IFN-λ1 levels and SLEDAI, anti-ds-DNA antibody and CRP, and there was also a negative relationship between IFN-λ1 levels and C3. CRP is an acute-phase protein known as a biomarker for inflammation, and has been traditionally used to detect and predict the outcome of infections, inflammatory, necrotic processes as well as monitor clinical disease activity together with efficacy of treatment [37,38]. Reports have displayed that CRP levels above 60 mg/l in febrile SLE patients without serositis almost always indicate infection; whereas in SLE alone, CRP levels are only moderately raised even in patients with very active disease [39]. In our study, the highest CRP value was 26.9 mg/l, and most CRP values were between 0 to 10 mg/l; therefore, in this study, CRP was a biomarker not indicating infection but monitoring disease activity. C3, one of complement components, participates in elimination of immune complexes through combination with immunoglobulin to disturb interaction of crystallizable fragment in space, and its reduction indicates disease activity. Therefore, by these results, we obtained the conclusion that IFN-λ1 may influence disease activity in SLE for a second time.

In patients with SLE, various manifestations make patients feel pain both physically and mentally. The most common clinical manifestations were arthritis (67%), malar rash (66%), nephritis (55%), and central nervous system (CNS) disease (27%) [40]. In consequence, we also assessed the correlation between IFN-λ1 and disease manifestations in patients with SLE; however, we did not distinguish the relationships among serum IFN-λ1 protein levels and alopecia, mucosal ulcer, malar rash, fever, chest affection, neurological disorder, anemia, thrombocytopenia and leucopenia. We discriminated that serum IFN-λ1 protein expression was significantly higher in patients with renal involvement and arthritis in comparison with patients without the above-mentioned disease manifestation as well as normal controls. In this case, we had evidence to infer that IFN-λ1 may involve in development of renal involvement and arthritis in SLE. Although our research did not observe an association between IFN-λ1 protein expression and other clinical manifestations, maybe this result was related to the limitation of small samples.

Since we had come to a decision that IFN-λ1 may participate in the pathogenesis of SLE, and has association with disease activity as well as progression of arthritis and renal disorder, we were interested in the mechanism by which IFN-λ1 played an important part in the development of SLE.

Chemokines are a group of molecules which have the ability to direct leucocytes to inflammatory sites to take part in immune response, influence maturation of kinds of immune cells, such as DC, T cell and stimulate monocytes, NK cells as well as T cell migration together with accommodate adhesion molecule expansion [20-22]. Chemokines IP-10, MIG and IL-8 are from the CXC family, and play an important role in the chronic inflammatory immune responses by recruiting leukocytes to inflammatory sites [25-27].

Besides, recent studies have reported that serum inflammatory chemokines IP-10 and MIG levels were elevated in SLE patients compared with normal controls. In addition, they had a positive relation with SLEDAI. Furthermore, in SLE patients with renal involvement, IP-10 and IL-8 levels were positively correlated with disease activity [29]. Beyond that, IP-10, IL-8 and MIG levels in cerebrospinal fluid (CSF) were significantly increased in neuropsychiatric SLE (NPSLE) patients compared with those with non-NPSLE and nonautoimmune diseases [41]. Another research team had calculated chemokine scores using several chemokines containing IP-10, MIG, IL-8 and other chemokines, finding that chemokine scores were significantly elevated in SLE patients versus RA patients and healthy donors and were correlated positively with SLEDAI scores and negatively with C3 levels. Compared with patients without lupus nephritis and those with inactive lupus nephritis, chemokine scores were increased in patients with active lupus nephritis. Elevated chemokine scores were also associated with the presence of cumulative organ damage and the occurrence of anti-Sm or anti-RNP autoantibodies [42]. Thus, elevation of chemokines could promote immune cells to migrate to inflammation site and take part in progression of inflammation by their cytotoxic effect or secretory inflammatory mediator in autoimmune diseases including SLE.

What is more, Pekarek V [36] had reported that IFN-λ1 could elevate mRNA levels of MIG, IP-10 and 'IFN-gamma inducible T-cell alpha chemoattractant' (I-TAC/CXCL11) from normal human PBMC. As a result, for exploring the function mechanism of IFN-λ1 in SLE, we detected the ability of IFN-λ1 to induce PBMC to secret MIG, IP-10 and IL-8 in patients with SLE, recognizing that in patients with SLE, IFN-λ1 had the ability to induce secretion of IP-10, MIG and IL-8. Meanwhile, it had its effects at three different concentrations with MIG levels increasing dose-dependently. Moreover, several concentrations of IFN-λ1 had a synergistic role with LPS in the production of chemokines IP-10, MIG and IL-8. Consequently, we deduced that IFN-λ1 presumably took effect in SLE by stimulating overexpression of chemokines IP-10, IL-8 and MIG associated with pathogenesis of SLE. In this case, it is reasonable to infer that after producing by a mount of abnormal PBMC, IFN-λ1 then stimulated PBMC to secret chemokines which could participate in the development of SLE through several different mechanisms. Therefore, this condition becomes a sort of vicious circle which is harmful to the disease situation of patients with SLE.

We know that bacterial infections may serve as environmental triggers for the development or exacerbation of SLE in genetically predisposed individuals [43], and that bacterial LPS plays an important role in the pathogenesis of SLE, such as, it could aggravate disease development by activating proliferation of B cells, production of autoantibodies and proinflammatory cytokines [44,45]. Moreover, Pawar RD *et al*. [46] reported that in nephritic MRL ^lpr/lpr ^mice, transient exposure to bacterial cell wall components LPS increased splenomegaly, production of DNA autoantibodies, serum IL-6, IL-12 as well as tumor necrosis factor (TNF) levels, and aggravated lupus nephritis (LN), which was a major complication of SLE and associated with high rates of morbidity [43]. In our study, we observed that there was a synergic effect of IFN-λ1 and LPS on the chemokines expression. Thus, we inferred that, except for the various effect of single LPS on the development of SLE and LN, together with the synergic effect of IFN-λ1 and LPS on the chemokines secretion, they could play a powerful effect on the inflammation process of SLE, and promote the disease aggravation in patients with SLE, especially with LN. Thus, we supposed that not only did IFN-λ1 participate in the renal involvement but also it played the pathogenic role by combining with the effect of LPS. Therefore, for patients with SLE accompanying LN, they should avoid bacterial infections in order to prevent disease progression.

At the same time, it is interesting to compare the role of different types of IFN in the pathogenesis of SLE. As is well known, Type I IFN (IFN-α, IFN-β) and Type II IFN (IFN-γ) are classic interferons, and play an important role in the pathogenesis of SLE. Some reports have displayed that compared to healthy and unrelated individuals, higher activity of IFN-α was found in the serum of both SLE patients and healthy relatives, and it was associated with autoantibodies to RNA-binding proteins and double-stranded DNA [47]. Levels of Type I IFN correlated with the presence of cutaneous manifestations, and positively with the SLEDAI score and anti-dsDNA levels and inversely with C3 levels [3]. Expression of IFN-γ was significantly increased in the PBMC of SLE patients compared with healthy controls [5], and its expression in urinary sediment was significantly higher in the active lupus nephritis than in inactive SLE and previous renal involvement. Among the SLE patients, there was a close correlation between expression of IFN-γ and the SLEDAI score [6]. Therefore, compared with the above mentioned research findings, we found that there was a lot of similarity with respect to the role of IFN-λ1 and Type I IFN as well as Type II IFN in the pathogenesis of SLE.

However, we must acknowledge some limitations of this study. The sample size of our study was small with 42 lupus patients and 25 controls, because a small sample size has a greater probability that the observation may happen to be especially good or bad, and statistical tests usually require a larger sample size to justify that the effect did not happen by chance alone. Moreover, owing to its cross-sectional design, it is difficult to establish the exact and definite causal relationships except association between IFN-λ1 and development of SLE from the collected data. Another disadvantage of such a study is that it can only identify a high proportion of prevalent cases of long duration. Patients who die soon or who recover quickly are less likely to be identified as diseased.

Meanwhile, because the mechanism of IFN-λ1 in the pathogenesis of SLE is very complicated, we hope there will be a lot of new findings about the role of IFN-λ1 in autoimmune diseases including SLE in the near future.

In conclusion, this preliminary study demonstrated that IFN-λ1 perhaps played a part in the pathogenesis of SLE and had a positive association with disease activity. Meanwhile, it was likely to refer to the development of renal disease as well as arthritis in SLE. Furthermore, we observed that IFN-λ1 probably played an important role in the pathogenesis of SLE by inducing production of chemokines IP-10, MIG and IL-8 which attended the progression of SLE. The present study of IFN-λ1 may offer new insight for future studies on pathogenesis of SLE and novel research targets for SLE therapy.

## Conclusions

The present study suggests that IFN-λ1, as one kind of interferon, may participate in the pathogenesis of SLE by modulating the expression of chemokines IP-10, MIG and IL-8, and IFN-λ1 may be related to disease activity in SLE. The findings of such studies will provide new insight into the pathogenesis as well as therapy of SLE, and will shed new light on the dysregulation of the immune system in autoimmune diseases.

## Abbreviations

AHA: anti-histone antibody; AnuA: anti-nucleosome antibody; C3: complement 3; CRP: C-reactive protein; DC: dendritic cells; ds-DNA: double stranded DNA; ESR: erythrocyte sedimentation rate; IFN-λ1: interferon-lambda 1; IL: interleukin; IP-10: IFN-inducible protein-10; LPS: lipopolysaccharide; IRF: interferon regulatory factor; LN: lupus nephritis; NK: natural killer; PBMC: peripheral blood mononuclear cells; rRNP: anti-ribosome ribonucleoprotein antibody; SLE: systemic lupus erythematosus; SLEDAI: systemic lupus erythematosus disease activity index; TNF: tumor necrosis factor.

## Competing interests

The authors declare that they have no competing interests.

## Authors' contributions

QW contributed to the experimental design, data acquisition, data analysis and interpretation, and the manuscript draft and edition. QY performed the data acquisition, analysis and interpretation of cell culture and ELISA. HS and YZ participated in data analysis and interpretation involving real-time PCR. EL contributed to data analysis and interpretation as well as the manuscript edition. All authors read and approved the final manuscript.
